# Rescue endoscopic retrograde cholangiopancreatography for duodenal perforation from maldeployed biliary stent

**DOI:** 10.1055/a-2852-7205

**Published:** 2026-04-29

**Authors:** Karthic Drishna Perumal, Steve Serrao, Hamza Shah, Erica Park, Somashekar G. Krishna, Raj Shah, Jordan Burlen

**Affiliations:** 1 24596 Mount Carmel Health SystemGrove City, OhioUnited States; 2 12306 The Ohio State University Wexner Medical CenterColumbus, OhioUnited States

**Fig. 1:**
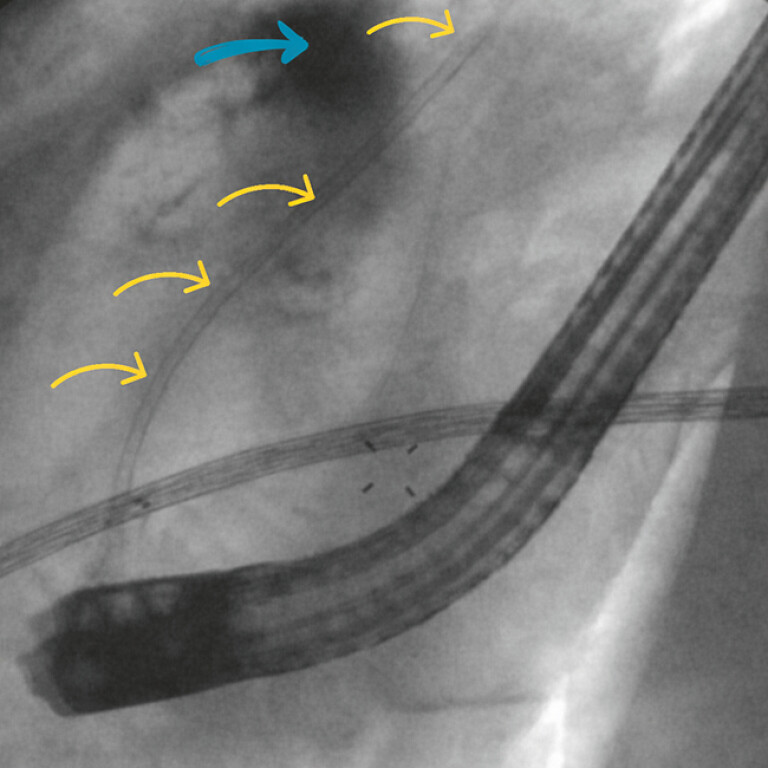
A fluoroscopic image of the malpositioned plastic biliary stent (yellow arrows) with associated filling defects (blue arrow).

Maldeployment of biliary stents with resulting duodenal perforation is an uncommon but potentially serious complication of endoscopic retrograde cholangiopancreatography (ERCP). Prompt recognition and timely response are critical to avoid surgical intervention. A 93‑year‑old man presented with 1 to 2 weeks of progressive jaundice. Initial computed tomography (CT) demonstrated the marked dilation of the common bile duct (CBD) and intrahepatic and pancreatic ducts. During index ERCP, deep cannulation was achieved; however, contrast injection produced poor opacification, minimal antegrade flow, and filling of a contained, dilated structure rather than a normal biliary tree ( Fig. 1 ). Multiple attempts to place a 7‑French × 9-cm plastic biliary stent failed to achieve drainage, raising concerns for false‑tract cannulation or extraluminal positioning. The procedure was terminated, and persistent abdominal discomfort prompted urgent repeat imaging.

**Fig. 2:**
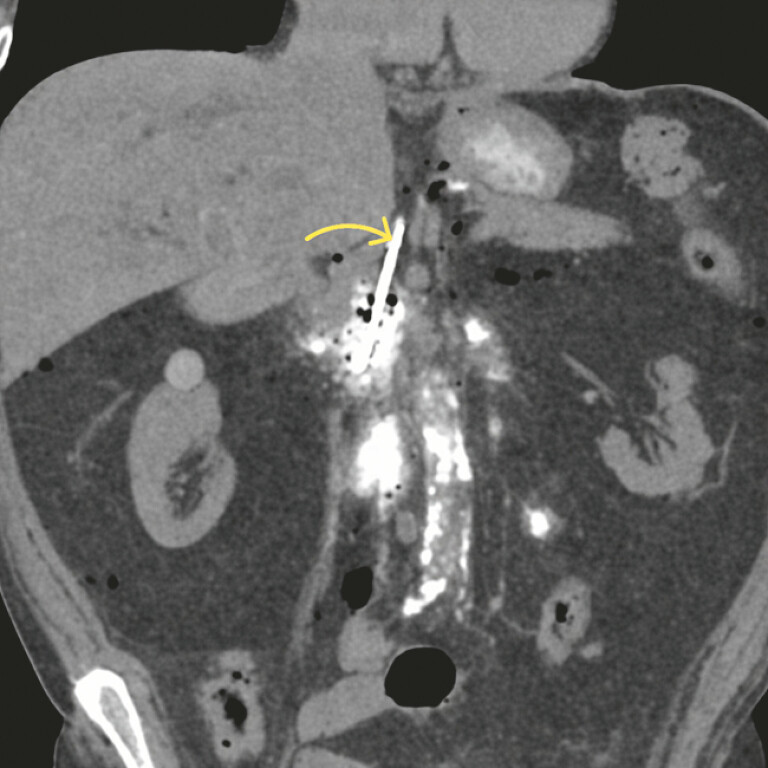
A CT image of the abdomen showing the malpositioned plastic biliary stent extending into the retroperitoneal space posterior to the pancreatic head with partial projection into the small bowel. CT, computed tomography.

**Fig. 3:**
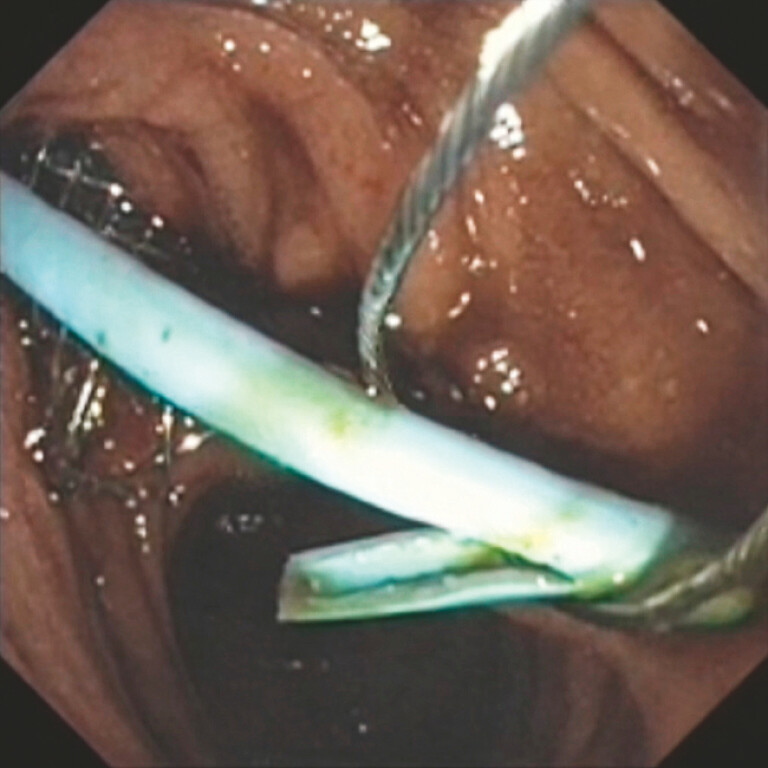
Removal of the maldeployed plastic biliary stent using the endoscopic snare technique.

**Fig. 4:**
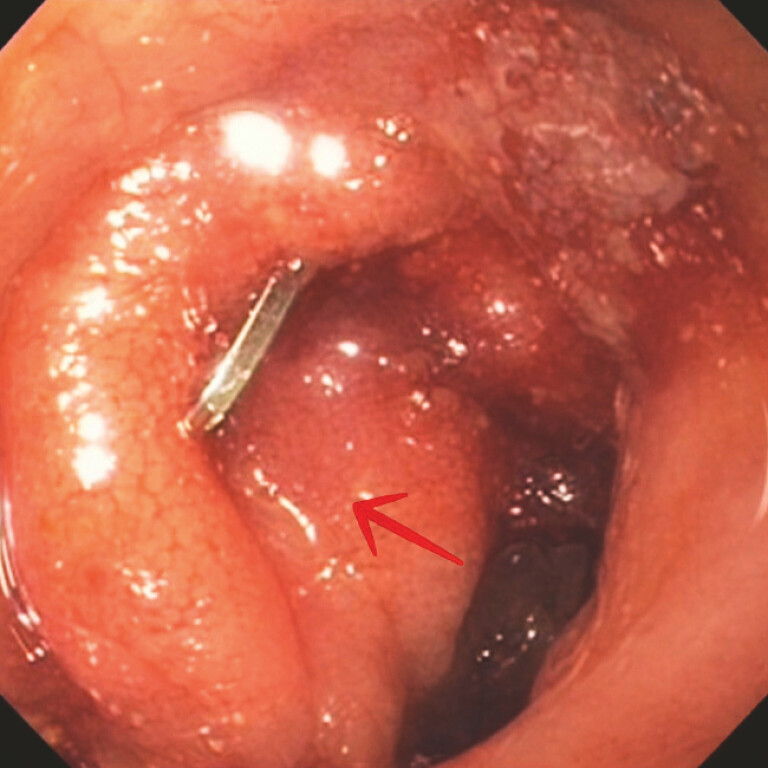
Successful closure of the mucosal defect with safe and effective deployment of an over-the-scope clip as highlighted by the red arrow.

CT revealed the plastic stent extending extraluminally behind the pancreatic head, with only a small portion projecting into the small‑bowel lumen ( Fig. 2 ). Scattered free intraperitoneal air and contrast extravasation tracked posterior to the pancreas along the second portion of the duodenum, with marked biliary dilation also present. These findings were consistent with stent maldeployment, retroperitoneal leak, and duodenal perforation. Given the patient’s stability, urgent rescue ERCP was performed. A 6‑mm duodenal perforation was identified just above the major papilla, with the stent protruding through the defect ( Fig. 3 ). The stent was removed, followed by selective pancreatic duct cannulation and deep biliary cannulation. Cholangiography demonstrated a 5‑mm distal CBD stricture with upstream dilation. Biliary sphincterotomy was performed, and a fully covered self‑expanding metal stent (10 mm × 60 mm) was deployed with immediate drainage ( Video 1 ). A 5‑French × 7‑cm pancreatic duct stent was placed prophylactically, and the perforation was closed using a single over‑the‑scope clip ( Fig. 4 ). Follow‑up CT showed no ongoing leak and appropriate stent positioning. This case highlights the effectiveness of advanced endoscopic rescue techniques in managing contained duodenal perforations.

Rescue ERCP with FCSEM stenting and over-the-scope clip closure successfully used to manage a maldeployed biliary stent causing a contained duodenal perforation in a 93‑year‑old man. ERCP, endoscopic retrograde cholangiopancreatography; FCSEM, fully covered self‑expanding metal.Video 1

Endoscopy_UCTN_Code_CPL_1AK_2AC

